# Family Alliance and Intergenerational Transmission of Coparenting in Gay and Heterosexual Single-Father Families through Surrogacy: Associations with Child Attachment Security

**DOI:** 10.3390/ijerph19137713

**Published:** 2022-06-23

**Authors:** Nicola Carone

**Affiliations:** Department of Brain and Behavioral Sciences, University of Pavia, Piazza Botta 11, 27100 Pavia, Italy; nicola.carone@unipv.it

**Keywords:** coparenting, single fathers, surrogacy, attachment security, extended family network, nonparental caregivers

## Abstract

Parents tend to internalize the coparenting model they experienced during childhood and enact it in their coparenting relationships as adults. These interactive patterns may, in turn, shape their children’s internal working models of attachment relationships. The present study recruited 31 gay and 28 heterosexual single-father families through surrogacy to examine family alliance quality and the mediating role of observed supportive and conflictual coparenting in the association between the coparenting quality single fathers experienced in their families of origin and the attachment security of their children. All single fathers lived in Italy, were cisgender and White, and had a child aged 6–12 years (*M* = 97.73 months; *SD* = 20.48; 47.5% girls) who they coparented with nonparental caregivers (i.e., 33 grandparents, 18 babysitters, 8 uncles/aunts). Families did not differ in family alliance dimensions based on fathers’ sexual orientation. Additionally, single fathers who experienced greater coparenting quality in their families of origin demonstrated lower levels of conflictual coparenting, which, in turn, were associated with greater child attachment security. In contrast, observed supportive coparenting did not mediate this relation. The results emphasize the need to reconceptualize the dyadic coparental unit in single-father surrogacy families to include extended family members and nonrelatives.

## 1. Introduction

Most single-father families are formed in the wake of parental separation or divorce. Less commonly, they arise following the death of the mother, when the mother lacks interest in parenting or loses custody due to neglect or abuse, or when children actively choose to live with their father [1]. Very recently, heterosexual, gay, and bisexual men have elected to become single parents of adopted children or children born through surrogacy—a practice whereby a woman (the “surrogate”) bears a pregnancy for the intended parent(s) with the intention of handing over the resulting child. These demographically small, but growing, family forms are comprised of so-called “single fathers by choice” or “elective single fathers” [2,3]. The present study involved families headed by gay or heterosexual single fathers through surrogacy. All fathers were residing in Italy, where surrogacy is banned (thus, individuals who wish to have children via this pathway must do so transnationally). While preliminary evidence suggests that gay and heterosexual single-father families through surrogacy demonstrate good parenting quality and have children with healthy adjustment [3,4], knowledge is lacking on triadic processes and coparenting (i.e., the processes by which two adults share leadership and provide mutual support when working together as parents) [5] in these families.

Triadic interactions and coparenting may seem irrelevant in single-father families, given the lack of a second parent and the father’s decision to parent alone [2]. However, it is not yet known whether single fathers through surrogacy indeed parent alone once their child is born, or whether they coparent with another adult within their social or family networks, in spite of their original intention. Previous research with single mothers who did not elect to parent alone (e.g., those who were teen mothers, got separated/divorced from the child’s biological father, were widowed) suggests that extended family networks (e.g., the child’s grandparents, aunts, or uncles) play an important role in childrearing, whether through indirect pathways (e.g., the provision of financial assistance) or through direct participation in childrearing activities [6,7]. In this vein, preliminary research conducted in Italy with single fathers through surrogacy (from which part of the present study sample was derived) shows that these fathers are generally affluent [2]. Therefore, it is likely that single fathers’ extended family networks contribute to childrearing directly, through activities such as assisting with school transportation and caregiving while the father is at work, instead of financially. In addition, ethnographic research highlights that nonrelatives (e.g., friends and neighbors) frequently provide emotional and instrumental support to single parents [7].

Coparenting scholars have highlighted that the coparenting construct is relevant for understanding family processes in diverse and single-parent families [8,9]. However, to date, no research has explored coparenting arrangements in single-father families through surrogacy, and it remains unknown whether single fathers actually coparent with an extended family member or nonrelative, and, if so, the extent to which this arrangement influences child adjustment. In the present study, child adjustment was examined in terms of attachment security.

### 1.1. The Family Alliance and Coparenting in Single-Parent Families

The advent of family systems theory and the subsequent increase in attention to triadic (e.g., mother–father–child) interactions—rather than dyadic (e.g., mother–child) interactions alone [10]—have led to increased research on coparenting. To the extent that coparenting is conceptualized as a triadic (i.e., whole family) level of analysis within the family system [5,11], it comprises varying dimensions of interactions. Supportive interactions describe those in which parents support and reinforce one another’s parenting activities, and unsupportive interactions describe inconsistent coparenting, conflict, and parents’ undermining of one another’s parenting efforts.

In parallel, empirical and clinical evidence shows that interaction quality within the family is associated with children’s social, emotional, and cognitive development (e.g., [12,13,14]). In this vein, the family alliance has been defined as the family’s ability to work together as a team during daily activities involving both parents and the child, such as during playtime, meals, or caretaking [15,16]. The family alliance has been identified as a key family-level process (i.e., a relational phenomenon that emerges from group-level interactions) that is critical to family dynamics. The family alliance model outlines four components of interactive family coordination: (a) the participation of all family members; (b) role organization; (c) focalization on a common interactive focus; and (d) affect sharing and empathy [15,16,17]. A family alliance may be categorized as “cooperative”, “conflicted”, or “disordered”, depending on the relative levels of cooperation, competition and conflict, and exclusion and chaotic interactions, respectively, within the triad. Conflicted and disordered family alliances are considered potentially problematic.

Of note, the family alliance cannot be derived from the observation of the separate mother–child, father–child, or mother–father dyads, or from the analysis of self-reported data [18]. Rather, the best way to capture its emergent properties is to observe the system “in action”—that is, through direct observation of triadic (or polyadic) interactions during free play or semi-standardized situations, such as the Lausanne Trialogue Play (LTP) [17,19]. The LTP is a standardized, observational, triadic situation designed to assess a family’s degree of coordination while completing a rather complicated task. Within this procedure, the child is incorporated as an active family member in different interactive contexts (i.e., being stimulated by one parent or by both, and watching them discuss). Additionally, the parents are tasked with acting out different roles (i.e., being active under observation, coordinating with the other parent). The LTP’s systematic assessment with coding grids evaluating nonverbal interactive dimensions has shown good criterion-related validity and construct validity. Additionally, the LTP is widely used for clinical and research purposes [15,17].

Previous research with heterosexual biological parent families has indicated that the family alliance is fairly stable from pregnancy to children’s toddler years, and predictive of child outcomes at the ages of 18 months and 5 years, particularly with respect to social skills, theory of mind, and understanding of inner states [12,20,21]. Other studies have linked unresolved conflict within the parental unit to several adverse child outcomes [22,23,24], whether due to the disruption of both parents’ relationships with the child (i.e., the so-called “spillover effect”, whereby parenting behavior becomes irritable and distant) or due to the child’s witnessing of hostile and competitive exchanges between parents.

Observation instruments and procedures, including the LTP, have rarely been used to assess the family alliance and coparenting in families other than those formed by two heterosexual parents through unassisted conception (for exceptions, see [25,26]). Additionally, although the LTP was developed to analyze triadic interactions up to middle childhood, most LTP studies have involved only infants and preschool children (e.g., [12,17,27,28]). Therefore, further LTP research is needed with school-age children, because, at this age, children develop more sophisticated strategies for controlling and self-regulating their behavior, become increasingly competent in communicating with parents about their internal states (thereby becoming better adjusted), and are more able to manage developmental challenges (thereby improving their engagement with parents and shared activities) [29]. In fact, child involvement and goal-directed partnership are the two interactive scales used in the LTP to assess the unique contribution offered by the child to triadic interactions.

Current knowledge about coparenting and childrearing assistance in single-parent families mainly derives from research with single mothers who did not elect to have a child and/or to parent alone (e.g., [6,30,31,32]). This research shows that the quality of the relationships these single mothers have with their coparents is associated with both maternal and child adjustment [31,32,33]. Mothers who report greater conflict with coparents regarding childrearing are more likely to have children with greater internalizing and externalizing difficulties than are mothers who report less conflict [33]. This line of research suggests that, if the relationships between single fathers and other adults with whom they coparent are positive, there may be benefits for child adjustment.

As the present study examined single fathers who were heterosexual or gay, previous research on the family alliance and coparenting across diverse family forms (with respect to parents’ sexual orientation) is also relevant. Studies with two-parent families have shown many similarities in parents’ management of coparenting-related challenges, regardless of their sexual orientation [34,35,36]. Nonetheless, they have also emphasized some unique and specialized ways in which gay and lesbian parents approach decisions about parenting roles and responsibilities. While heterosexual couples tend to specialize more than gay and lesbian couples (i.e., heterosexual mothers tend to engage in more unpaid childcare labor while heterosexual fathers tend to work more outside the home), gay and lesbian couples tend to be more equitable in their parenting tasks and roles and more satisfied with their division of responsibilities (e.g., [26,37,38,39]). Overall, irrespective of parents’ sexual orientation, supportive coparenting has been shown to be associated with better child adjustment [40]. In terms of the family alliance, no study has been conducted with gay fathers. However, some evidence from lesbian mother families shows similar functional family alliances in lesbian mother and heterosexual parent families [25,26]. Whether these results also extend to gay and heterosexual single fathers through surrogacy remains unknown.

### 1.2. Associations between Coparenting Experienced in the Family of Origin and Child Attachment Security through Observed Coparenting

The intergenerational transmission of parenting holds that parents’ own experiences as a child influence their childrearing practices and attitudes [41,42]. What is transmitted across generations, however, is not only a behavioral style, but also a more complex structure of the self that impacts one’s relationship with one’s own child, at both conscious and unconscious levels, and is likely activated during parent–child interactions [43]. Similarly, parents internalize the coparenting model they experienced during childhood and express this as an interaction style in their coparenting relationships as adults [44,45]. Therefore, individuals who observed a competent parenting partnership in their family of origin likely have a more functional framework for their own coparenting [45].

To date, only a few studies have examined the associations between relationships in parents’ families of origin and actual coparenting quality. These studies have all been conducted with heterosexual biological parent families, in which the coparents share (or shared, in the case of divorce) a marital relationship. For example, among 47 heterosexual intact couples, McHale [46] found that mothers’—but not fathers’—relationships with their own parents were related to spousal discrepancies in warmth and investment during interactions with their infant children. Additionally, in a study with 101 heterosexual couples, Van Egeren [45] showed that fathers who perceived their own parents as having maintained a successful coparenting relationship were more likely to rate their own coparenting positively when their children were aged 1, 3, and 6 months. The Van Egeren [45] study suggests that models for negotiating parental disagreements and providing coparental support may be particularly valuable for heterosexual men. It further suggests that heterosexual men tend to experience a general relationship quality that links multiple relationships in their lives (i.e., with their coparent and their family of origin), in contrast to heterosexual women, who tend to evaluate particular relationships with greater specificity [47]. It is not yet known whether coparenting in a parent’s family of origin makes a unique contribution to the parent’s own coparenting within triadic interactions in diverse family forms, such as single-father families through surrogacy.

Looking at the effects of coparenting on the parent–child relationship, a family systems perspective on attachment [48] suggests that family functioning at the triadic level may directly influence functioning at the dyadic level (parent–child). Nonetheless, the association between coparenting and the child–parent attachment relationship has rarely been examined. Additionally, again, our current knowledge derives from heterosexual biological parent families. In this family type, Newland et al. [49] found that a higher quality of father-reported coparenting was associated with higher secure-base behavior in children. Additionally, Caldera and Lindsey [50] found that competitive coparenting was associated with less secure infant–mother and infant–father attachment relationships. Another study by Perez et al. [51] showed that cooperative triadic interactions were related to more secure attachment representations among preschool children. Similarly, Brown et al. [52] documented a positive association between supportive coparenting and infant–father attachment security, while finding a nonsignificant association between supportive coparenting and infant–mother attachment security. Support for the abovementioned results was provided by a meta-analysis by Teubert and Pinquart [53], which revealed insecure child–parent attachment among children experiencing more negative coparenting.

Of note, the different results for mothers and fathers suggest that family relations may have divergent effects on the emergence of infant–mother and infant–father attachment relationships. This theory is aligned with studies reporting that family relations have a greater effect on fathers than on mothers [54,55]. However, as the abovementioned studies involved heterosexual two-parent families (in which mothers tend to be more engaged than fathers in childcare), it cannot be determined whether the results reflect the effects of parent gender or caregiving role [35]. In this vein, research with single-father families through surrogacy, in which fathers are likely to be the primary caregivers, is essential to gain further insight into this question.

Multiple explanations for how coparenting might affect the child–parent attachment relationship exist, as also noted by Brown et al. [52]. One possibility is that coparenting directly shapes children’s internal working models of attachment relationships [56]. Indeed, interparental discord is thought to promote feelings of helplessness and self-blame [57] that may be reflected in child–parent attachment relationships or children’s representations of these relationships. In this vein, discord around childrearing may be especially likely to affect the child’s attachment system [58], whereas support and harmony between parents may promote greater security in child–parent attachment relationships. Overall, it stands to reason that children who experience cooperative, coordinated, and supportive coparental interactions may perceive their parents as secure and trustworthy caregivers to whom they can return in times of distress, danger, or illness. In contrast, children who are exposed to discordant, conflicted, and competitive parental interactions may experience feelings of insecurity and uncertainty towards each parent [50]. Second, according to the emotional security hypothesis [59], children’s chronic exposure to undermining behaviors between caregivers may contribute to emotional insecurity and subsequent difficulty regulating their own emotions [60].

Third, Belsky’s [61] process model of the determinants of parenting suggests that childrearing support from extended family networks or nonrelatives might represent a double-edged sword for single fathers, as recently also suggested by research on single mothers with grandparent childcare arrangements [62,63,64]. On the one hand, such support may help fathers attend to their children while, at the same time, allowing them to focus on their performance at work. This may enhance single fathers’ self-esteem and parental efficacy, with positive implications for parenting. On the other hand, tension and conflict in the father’s extended family/nonrelative childcare networks may become a source of stress and disruption. In cases where the nonparental caregiver is the child’s grandparent, uncle, or aunt, single fathers may re-experience unresolved family conflicts that exert a negative effect on their coparenting; alternatively, in cases where the nonparental caregiver is a friend or babysitter, the single father and the caregiver may have experienced very different coparenting styles in their families of origin, resulting in increased difficulty sharing coparenting values, styles, and practices.

To date, there has been no research on the effect of coparenting between single fathers and nonparental caregivers on child attachment security. Based on the research and theories reviewed above, the present study preliminarily examined who single fathers identified as coparents and whether family alliance dimensions (including coparenting) and child attachment security differed across family type and child gender, as well as whether there were differences between gay and heterosexual single fathers in the coparenting quality they experienced in their families of origin. Finally, the study investigated the intergenerational transmission of coparenting and its effect on the attachment security of single fathers’ school-age children born through surrogacy. Specifically, it was hypothesized that single fathers who experienced higher coparenting quality in their families of origin would have children with higher levels of attachment security, and this relationship would be mediated through higher supportive coparenting and lower conflictual coparenting during the LTP.

## 2. Materials and Methods

### 2.1. Participants

The present study comprises part of a larger project on parenting and child adjustment in single-father families through surrogacy [3,4,65]. However, data presented here have not been published before. Participants were 31 gay single-father families and 28 heterosexual single-father families through surrogacy. All families lived in Italy and had a child aged 6–12 years. In families with more than one child in the relevant age range, the oldest child was studied. All fathers self-identified as cisgender, were White, and lived alone with their target child. Although single-father families were not recruited on the basis of the type of surrogacy practiced (i.e., genetic vs. gestational), all used gestational surrogacy (involving the father’s sperm, an egg donor, and a gestational carrier). All families were required to have a nonparental caregiver who frequently (i.e., at least three times per week) spent time with the child. Then, each single father was asked to identify the second most important person (if any) who assisted in childrearing. In this category, the fathers identified 33 grandparents, 18 babysitters, and 8 uncles/aunts.

To be included in the larger project, single fathers (a) had to self-identify as gay or heterosexual; (b) must have decided to undertake parenting alone; (c) could not have cohabited since the target child’s birth; (d) could not have been involved in a non-cohabiting relationship lasting longer than 6 months; (e) needed to have a target child aged 6–12 years who had been conceived through surrogacy. As single-father families are an extremely difficult-to-reach population, multiple recruitment strategies were used: (a) the researchers posted online advertisements on the websites of single-parent groups (*n* = 18, 30.5%); (b) participants passed information about the study to friends, colleagues, and acquaintances who fit the study criteria and/or disseminated information about the study through social media (*n* = 36, 61.0%); and (c) an association of same-sex parents distributed information about the study via their mailing list (*n* = 5, 8.5%). Table 1 presents families’ demographic characteristics.

### 2.2. Procedure

The study was conducted according to the guidelines of the Declaration of Helsinki, and was approved by the Ethics Committee of the Department of Developmental and Social Psychology of Sapienza University of Rome (protocol 245/2016; Title: “Parent–Child Relationship and Child Adjustment in Single-Father Families Formed Through Surrogacy”). Written informed consent was obtained from all adult participants (i.e., fathers and nonparental caregivers). Parents also consented for their child to participate and the nonparental caregiver to be contacted. Verbal assent was gained from children. Each participant was reminded that their responses would be confidential and that participation in all or part of the study could be terminated at any time; such information was conveyed to the children in an age-appropriate manner, both before and during their participation. Families were assessed at home by a researcher trained in the study techniques, and no compensation was offered to participants. Data were collected between November 2016 and May 2019.

### 2.3. Measures

#### 2.3.1. Coparenting in the Family of Origin

Each father completed the 12-item questionnaire created by Stright and Bales [44] to assess coparenting in their family of origin during childhood. The frequency with which parents displayed supportive (6 items: e.g., “My parents listened to one another when one of them had something to say about me”) and conflictual (6 items: e.g., “My parents criticized each other’s parenting”) coparenting behaviors was rated on a 5-point scale ranging from 1 (*never*) to 5 (*always*). Overall scores for coparenting quality in fathers’ families of origin were calculated by reverse coding the conflictual items and averaging all items, with higher scores indicating higher coparenting quality. Cronbach’s alphas were 0.87 and 0.89 for gay and heterosexual single fathers, respectively.

#### 2.3.2. Observed Family Alliance and Coparenting

The family alliance and coparenting dynamics were observed during the LTP [17,19]. The LTP is a semi-standardized role-play situation designed to systematically observe how a family of three handles triangular interactions as they move through four possible relational configurations. In the present study, the relational configurations included: dyadic father–child play in the presence of the nonparental caregiver; dyadic nonparental caregiver–child play in the presence of the father; father–nonparental caregiver–child triadic play; and father–nonparental caregiver discussion of this task while the child watches. Although the LTP was originally developed and conducted as a laboratory procedure [17,19], in the present study it was adapted for the home setting, in order to reach as many families as possible (for more information on LTP conducted in the home setting, see [62,66]).

Given the children’s age, each family was invited to cooperate and work together to plan a birthday party as they would usually do. The father, the nonparental caregiver, and the child sat at a round table, with their body positions forming a triangle. Interactions were videotaped using two cameras—one recording the father and the nonparental caregiver from the front and the other recording the child. Participants were instructed that the duration of the entire task would be approximately 12–15 min (according to the standard duration of triadic free play in naturalistic conditions with children older than 18 months) [17,19]. Subsequently, they decided how long each scenario would last. The mean length of the entire task was 13:31 min (range: 10:58–15:02).

Each video was analyzed using the Family Alliance Assessment Scale (FAAS), version 6.3 [17]. The FAAS includes 15 scales (i.e., *Postures and Gazes*, *Inclusion of Partners*, *Role Implication*, *Structure*, *Co-construction*, *Parental Scaffolding*, *Family Emotional Warmth*, *Validation*, *Authenticity*, *Interactive Mistakes During Activities*, *Interactive Mistakes During Transitions*, *Coparental Support*, *Coparental Conflict*, *Child Involvement*, *Child Goal-Directed Partnership*) measuring 7 principal interactive functions (i.e., *participation*, *organization*, *focalization*, *affect sharing*, *timing/synchronization*, *coparenting subsystem*, *child subsystem*). Each scale assesses the triadic interaction according to a 3-point scoring system, as follows: 0 (*inappropriate*), 1 (*moderate*), and 2 (*appropriate*). Scores on the first 11 scales may be summed to generate a family alliance score, ranging from 0 to 22. A single reliable coder coded all videos. To test interrater reliability, 25% of the videos were coded by a second reliable coder, who was unaware of the fathers’ sexual orientation (*Cohen’s ĸ* = 0.81 and 0.72 for the family alliance; overall, 15 scale scores, respectively, *p* < 0.001).

#### 2.3.3. Child Attachment Security

Children completed the 15-item Security Scale Questionnaire [67], which was used to assess their perception of attachment security to their father, using Harter’s [68] “Some kids… Other kids…” format (e.g., “Some kids find it easy to trust their dad BUT Other kids are not sure if they can trust their dad”). For each item, respondents are asked to indicate which statement is more characteristic of them and whether the statement is really true (1) or sort of true (4) for them. The scale generates a total score of attachment security by averaging the item scores, with higher scores indicating higher levels of perceived attachment security. In the present study, items were read aloud to the youngest children (aged 6–7 years), to ensure that they understood the questions. The reliability and validity of the SSQ have been assessed in both child and adolescent samples, showing moderate stability over time [69] and convergence with observations of children’s interactions with parents [67]. Cronbach’s alphas were 0.80 and 0.78 for children of gay and heterosexual single fathers, respectively.

### 2.4. Data Analysis

All analyses were performed using the statistical software R [70]. Descriptive statistics were used to report figures such as percentages, means, and standard deviations of sociodemographic variables, whereas correlations were used to report associations between children’s and fathers’ characteristics and the study variables. Subsequently, one analysis of variance (ANOVA) was performed to analyze differences in coparenting quality in fathers’ families of origin between gay single fathers and heterosexual single fathers. Additionally, a further ANOVA and one multivariate analysis of variance (MANOVA) were performed to explore differences in child attachment security and each family alliance dimension (including the continuous total score of family alliance), respectively, across family type and child gender.

Finally, one parallel mediation model was performed to examine the intergenerational transmission of coparenting and its effect on child attachment security, computing 95% confidence intervals with bootstrap percentiles and 5000 resamples, as recommended by Hayes [71]. Coparenting quality in the family of origin was entered as the predictor and observed supportive coparenting and observed conflictual coparenting were entered as mediators. When demographic variables were significantly associated with the predictor, mediators, and/or outcome, they were included as covariates in the mediation analysis. A post hoc Monte Carlo power simulation was computed to obtain the statistical power of the results for the indirect effects, using the shiny and MASS add-on R packages [72].

## 3. Results

### 3.1. Descriptive Analyses

Table 1 presents participants’ sociodemographic factors, and Table 2 displays the associations between children’s and fathers’ demographic factors, child attachment security, coparenting quality in fathers’ families of origin, observed coparenting, and the observed family alliance, by family type. Given the wide age range and the significant associations between child age and child attachment security, child age was entered as a covariate in the parallel mediation model.

### 3.2. Who Do Single Fathers Identify as Coparents?

As shown in Table 1, there were no differences among gay and heterosexual single fathers in the nonparental caregiver category they identified as coparents, *χ^2^*(2) = 1.270, *p* = 0.530. Specifically, in gay single-father families, more than half of the identified coparents (*n* = 17, 54.8%) were the child’s grandparent, of whom 14 were the single father’s mother and 3 were the single father’s father; approximately one-third (*n* = 11, 35.5%) were the child’s babysitter; and the remaining 3 (9.7%) were the child’s uncle (*n* = 1) or aunt (*n* = 2). In heterosexual single-father families, most coparents (*n* = 16, 57.1%) were the child’s grandparent, of whom 15 were the single father’s mother and 1 was the single father’s father; one-quarter (*n* = 7, 25.0%) were the child’s babysitter, and the remaining 5 (17.9%) were the child’s aunt.

### 3.3. Differences in Coparenting Quality in Fathers’ Families of Origin across Fathers’ Sexual Orientation

The ANOVA indicated that gay single fathers and heterosexual single fathers did not experience significantly different coparenting quality in their families of origin, *F*(1,57) = 0.257, *p* = 0.614, *η*_p_^2^ = 0.004 (*M* = 3.73, *SD* = 0.40; *M* = 3.67, *SD* = 0.50, respectively).

### 3.4. Differences in Child Attachment Security and Family Alliance across Family Type and Child Gender

The ANOVA indicated that children of gay single fathers and children of heterosexual single fathers showed similar levels of attachment security, *F*(1,55) = 0.317, *p* = 0.860, *η*_p_^2^ = 0.001 (*M* = 3.13, *SD* = 0.35; *M* = 3.11, *SD* = 0.35, respectively). Additionally, there were no differences in attachment security across child genders, *F*(1,55) = 0.586, *p* = 0.447, *η*_p_^2^ = 0.011 (girls: *M* = 3.08, *SD* = 0.36; boys: *M* = 3.15, *SD* = 0.34), and the interaction between family type and child gender was not significant, *F*(1,55) = 0.925, *p* = 0.340, *η*_p_^2^ = 0.017.

Regarding the family alliance analyzed in its interactive functions, timing/synchronization, and subsystems, and observed during the LTP, the MANOVA indicated that neither family type, Wilks’ λ(16,40) = 0.727, *p* = 0.536, *η*_p_^2^ = 0.273, nor child gender, Wilks’ λ(16,40) = 0.739, *p* = 0.590, *η*_p_^2^ = 0.261, had a significant effect. Additionally, the interaction between family type and child gender was not significant, Wilks’ λ(16,40) = 0.784, *p* = 0.787, *η*_p_^2^ = 0.216. Given their nonsignificant effects, family type and child gender were not included in the mediation analysis. Table 3 shows the complete statistics.

### 3.5. Parallel Mediation of Observed Supportive and Conflictual Coparenting in the Association between Coparenting Quality in Fathers’ Families of Origin and Child Attachment Security

**Figure 1:**
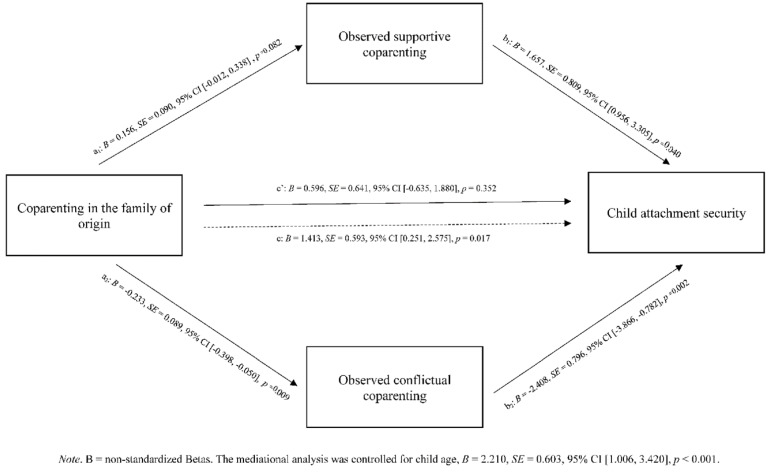
Parallel mediation of observed supportive and conflictual coparenting in the association between coparenting in the families of origin and child attachment security (N = 59).

The parallel mediation analysis with confidence intervals computed using the bootstrap percentiles method and 5000 resamples indicated that the total effect was significant, point estimate = 1.413, *SE* = 0.593, 95% CI [0.251, 2.575], *p* = 0.017. Specifically, as shown in Figure 1, the indirect path from coparenting quality in the family of origin through LTP observed conflict to child attachment security was significant, point estimate = 0.561, *SE* = 0.269, 95% CI [0.084, 1.121], *p* = 0.037, suggesting that the greater coparenting quality single fathers experienced in their families of origin resulted in lower levels of conflictual coparenting observed during the LTP, which, in turn, was associated with higher levels of child attachment security. Conversely, the indirect path from coparenting quality in the family of origin through LTP observed support to child attachment security was not significant, point estimate = 0.259, *SE* = 0.236, 95% CI [−0.022, 0.867], *p* = 0.273. A Monte Carlo power analysis for indirect effects with observed LTP support and observed LTP conflict as mediators showed a low power of 15% and a moderate power of 51%, respectively (based on a 95% CI).

## 4. Discussion

The present study observed the family alliance during triadic interactions within the LTP procedure, and examined the influence of intergenerational transmission of coparenting on child attachment security among gay and heterosexual single fathers through surrogacy with school-age children. For descriptive purposes, it is important to note that, although all single fathers resided alone with their child(ren), this did not mean that extended family members and other adults were not intricately involved in coparenting. In fact, all single fathers identified a coparent (i.e., their child’s grandparent, babysitter, uncle, or aunt) who not only spent significant time with their child, but also assisted them in childrearing. In parallel, the finding that none of the 17 single fathers with a partner identified their partner as a coparent might be explained by the fact that, to be included in the study, the single fathers could not have been involved in a cohabiting relationship for longer than 6 months. Thus, the relatively short duration of their romantic relationships may have influenced their decision not to involve their partner in coparenting activities.

While coparenting in single-father families through surrogacy may seem to contradict the definition of “single fathers by choice” [2], some single fathers may have to revise their initial intention to parent alone, particularly if they work full-time and need to balance their parenting role with professional and social roles. To date, only one cross-sectional study has examined parenting, family functioning, and child adjustment in families headed by “single fathers by choice” [3,4,65]. Thus, further longitudinal research is needed to clarify changes and discrepancies (if any) in single fathers’ intention to have a child and parent alone. Such research could also shed light on whether single fathers by choice attribute different meanings to conceiving and parenting alone, and whether the involvement of a nonparental coparent derives from the actual difficulty they experience when attempting to parent alone.

Single fathers’ use of coparenting networks points to the need to examine how single fathers and nonparental caregivers coordinate and interact with children during triadic interactions. In the present study, on average, all family members were appropriately interactive and interested in each other (participation), as well as appropriately able to take turns and/or to attribute differentiated roles according to the aim of the interaction (organization) in the LTP. Additionally, each family member’s attention and gestures were appropriately focused on the co-construction of the birthday party planning (focalization) and, when there was joint attention between them, the emotional expressions of all partners were attuned, and mutual empathy circulated between them (affect sharing).

In terms of timing and synchronization, there were few communication mistakes (misunderstanding, miscoordinations) during interactions. When these occurred, they were repaired quickly. Furthermore, when members transitioned from one part of the game to another, interactions were reorganized smoothly, with quick and resolved negotiations. Finally, regarding the coparental and child subsystems, single fathers and nonparental caregivers showed, on average, moderate support and low conflict while coparenting; additionally, children were moderately involved and showed a moderate goal-directed partnership. Although it was not possible to compare these results with the normative Lausanne sample provided by Favez et al. [17], given the significant difference in children’s age, a closer look at the coding manual reveals that each interactive function was executed, on average, in an appropriate manner, regardless of the single fathers’ sexual orientation.

With respect to the hypothesized influence of intergenerational transmission of coparenting on child attachment security, it was found that single fathers who experienced greater coparenting quality in their families of origin demonstrated lower levels of observed conflictual coparenting, which, in turn, was associated with greater child attachment security. This is consistent with the intergenerational transmission of (co)parenting model [41,42,44,45] and the determinants of parenting [61] theory, indicating that parents inevitably bring models from their families of origin to family interactions, and that parents’ developmental histories influence their parenting.

The finding that coparenting conflict among single fathers and nonparental caregivers during the LTP was associated with parent–child relationship difficulties (i.e., lower levels of child attachment security) echoes prior research conducted with biological intact and separated heterosexual parent families and single-mother families [32,73,74]. According to the emotional security hypothesis [59], children’s internalized representations of coparental relations and response processes, which develop over time, have implications for their long-term adjustment. In this vein, it cannot be excluded that children’s ratings of child–father attachment security were affected by previous experiences of single father–nonparental caregiver conflict, also considering that the youngest participating child was 6 years old and had likely “accumulated” some experiences of such a coparenting network arrangement. However, the assessment of child attachment security took place at the same time as the observation of coparenting quality, and, importantly, the length of time that the nonparental caregiver had been engaging in coparenting activities was not assessed. This latter variable should be included in future research on coparenting network arrangements in single-parent families, in order to confirm this idea.

Conversely to observed conflictual coparenting, observed supportive coparenting was not a significant mediator of child attachment security, and it was not significantly associated with single fathers’ coparenting experiences in their families of origin. This finding contrasts with previous evidence showing that mothers’ recollections of supportive coparenting in their families of origin are positively associated with their supportive coparenting [44], and that observed supportive coparenting is associated with greater attachment security in the infant–father attachment relationship [52]. However, the different family types (single-father surrogacy families in the present study vs. heterosexual two-parent families through unassisted conception in previous studies), parent gender, child age, and measures used to assess coparenting may have extensively contributed to the discrepant findings.

In a similar vein, the significant and nonsignificant effects of conflictual and supportive coparenting, respectively, may be a peculiar consequence of the coparenting arrangement in single-father families. From an intergenerational perspective, it stands to reason that, although both positive and negative family interaction patterns in parents’ families of origin may be transmitted across generations, single fathers may be more accurate in remembering negative coparenting interactions because these are still salient and unresolved (to some extent), and thus easier to report. During the LTP paradigm in the present study, single fathers may have re-experienced past family conflicts—or a trace of them—while interacting with the nonparental caregiver. This seems especially plausible, since out of the 59 nonparental caregivers, 41 (69.5%) belonged to the single fathers’ families of origin (i.e., a parent, brother, or sister). Similarly, in line with Belsky’s [61] process model of the determinants of parenting and the darker role played by social support, in the 18 cases where the nonparental caregiver was the child’s babysitter, the babysitter’s family of origin may have had different coparenting values, styles, and practices that emerged during the LTP and re-activated the single fathers’ memories of previous coparenting conflicts.

Several limitations of the study merit attention. First, the data were cross-sectional, and the parallel mediation model presupposed a unidirectional association between the quality of coparenting experiences in fathers’ families of origin, the observed coparenting relationship, and child attachment security. Longitudinal research would allow for potential bidirectional associations to be explored, as well. For example, it is plausible that greater attachment security in children may contribute to more supportive and less conflictual coparenting, as the secure child–father attachment relationship may allow fathers to feel more confident and efficient as parents, and thus to perform better as coparents, regardless of their memories of coparenting in their families of origin. Second, only single fathers’ memories of childhood coparenting experiences were collected, with the risk of retrospective reporting bias. This also implies that nonparental caregivers’ memories of their past coparenting experiences were ignored, and thus their contributions to actual coparenting interactions were not considered.

Third, in keeping with previous research, other variables may contribute to the link between coparenting in fathers’ families of origin, observed coparenting, and child attachment security––most notably fathers’ personality, psychosocial adjustment, parenting quality, gender role beliefs, and prenatal coparenting representations, as well as child temperament (e.g., [27,44,66,75,76,77]). Fourth, single fathers were not asked to indicate the specific childrearing activities in which nonparental caregivers participated, and the extent of this involvement. However, as fathers were asked whether there was a second most important person who assisted in childrearing, it is very likely that single fathers considered themselves the primary caregivers, also considering that all fathers lived alone with their child(ren). Finally, multiple sources were used to recruit as many families as possible. However, the final sample size was quite small; therefore, the results of this study should be interpreted with caution and need replication before firm conclusions can be drawn. In a similar vein, because of the relatively small number of cells in each nonparental caregiver category (i.e., grandparents, uncles, aunts, babysitters), it was not possible to examine whether the findings varied depending on the relation of the coparent to the single father–child dyad. However, the literature does not suggest differential outcomes based on coparent’s identity [32].

Notwithstanding these limitations, the study also has a number of strengths. It contributes to the sparse literature examining the relations between family processes across generations. In addition, it is unique in exploring the family alliance and coparenting dynamics in single-father families through surrogacy, which represent a small but growing family form in which diverse nonparental caregivers may participate in coparenting [1]. The application of a standardized, fine-grained instrument (i.e., the LTP) to observe triadic interactions represents a further strength, as this allowed for the consideration of children’s contribution to family dynamics. This is particularly relevant, since children’s behavior may significantly impact their caregivers [78] and, in middle childhood, children become increasingly competent in self-regulation and conflict management with parents/caregivers during shared activities [29], as required by the LTP. Furthermore, use of the LTP procedure is a further strength, given the triadic nature of the coparenting construct [5,11] and the subsequent need to observe each actor in the coparenting relationship (including the nonparental caregiver and the child), instead of relying on only single fathers’ reports of coparenting quality.

In terms of theoretical and clinical implications, the present study emphasizes the importance of the coparenting subsystem for child adjustment in families in which a nonparental caregiver is involved. In this vein, the results confirm both the specificity and the universality of coparenting as a key aspect of family functioning across diverse family forms. For practitioners working with single-father surrogacy families, the results may enlarge their understanding of the different forces (including nonparental caregivers) that affect child development. Although the study findings must be replicated, practitioners may find it useful to encourage single parents and nonparental caregivers to explore the models of coparenting they observed in their families of origin, as these models may affect their own coparenting practices. These theoretical and clinical implications are not limited to only single-father families, but extend to all families, regardless of parents’ gender, sexual orientation, and number, and children’s conception background.

Furthermore, the present study contributes to the emerging literature on the role of relational networks in children’s socioemotional development from the combined perspective of attachment and coparenting theories [59,62]. It demonstrates that the degree to which children become securely attached to their father may at least partly depend on the quality of the father–nonparental caregiver coparenting relationship. The study highlights that, to unravel the mechanisms underlying the development of the child–father attachment relationship in single-father families through surrogacy, we must look beyond the father–child dyad. This is consistent with the family system perspective of attachment relationships [48] and suggests that future studies aimed at identifying the precursors of children’s socioemotional development in single-father families through surrogacy should adopt a longitudinal design without an exclusive focus on paternal behavior.

It is important to focus on parents’ experiences in their families of origin not only because these experiences may affect later family relationships [41,42,44,45], but also because they may help to identify whether—and to what extent—parents have revised or created new models of coparenting to replace those of their childhood. Finally, while previous research with heterosexual biological two-parent families has found that women are more accurate in their reporting of family coparenting interactions than are men (e.g., [44]), the present study suggests that single fathers may provide helpful insights into family coparenting dynamics. In light of the different family configurations included in this and previous research, it cannot be excluded that parents’ caregiving role is a more powerful trigger for memories of family interactions than is gender during daily parenting activities [35], given that single fathers are primary caregivers, similar to many heterosexual mothers in two-parent families.

## 5. Conclusions

Family theorists and practitioners emphasize that family members are influenced by experiences and models from their families of origin [41,42,45]. The present study found that the quality of coparenting observed during triadic interactions was influenced by single fathers’ memories of interaction patterns in their families of origin, particularly with respect to conflictual coparenting; this relation, in turn, was associated with children’s attachment security during middle childhood. Overall, the study also indicated that, on average, single-father families through surrogacy interacted cooperatively and performed the LTP triadic interactive functions appropriately.

By definition, single fathers through surrogacy opt to have children and to parent alone. However, this does not preclude the possibility that extended family members and nonrelatives will contribute to childrearing. As a consequence, the coparenting framework must be extended to include “the relevant people in the family network and accept unconventional family shape” (p. 25, [79]). This is in recognition of the fact that the traditional definition of a coparent by marriage or a cohabiting romantic relationship (as usually applies in heterosexual biological two-parent families) must be stretched to understand the effect of triadic interactions and coparenting in single-father families through surrogacy.

## Figures and Tables

**Table 1:** Descriptive statistics of sociodemographic factors by family type (*N* = 59).

	Gay Single-Father Families (*n* = 31)	Heterosexual Single-Father Families (*n* = 28)	*χ^2^* (*df*)	*p*	
Child gender			0.169 (1)	0.681	
Boy	15 (48.4%)	16 (57.1%)			
Girl	16 (51.6%)	12 (42.9%)			
Number of siblings			0.052 (1)	0.819	
0	24 (77.4%)	20 (71.4%)			
1	7 (22.6%)	8 (28.6%)			
Family residence			0.021 (2)	0.990	
Northern Italy	13 (41.9%)	12 (42.9%)			
Central Italy	16 (51.6%)	14 (50.0%)			
Southern Italy	2 (6.5%)	2 (7.1%)			
Father race/ethnicity (White)	31 (100%)	28 (100%)	0.000 (1)	1.000	
Father educational attainment			1.202 (2)	0.548	
Undergraduate degree	6 (19.4%)	5 (17.9%)			
Master’s degree	16 (51.6%)	18 (64.2%)			
Post-doctoral degree	9 (29.0%)	5 (17.9%)			
Father work status			0.051 (1)	0.821	
Full-time	26 (82.9%)	25 (83.3%)			
Part-time	5 (17.1%)	3 (16.7%)			
Father relationship status			1.960 (1)	0.161	
Single	25 (80.7%)	17 (60.7%)			
In a relationship	6 (19.3%)	11 (39.3%)			
Nonparental caregivers involved in coparenting			1.270 (2)	0.530	
Child’s grandparent	17 (54.8)	16 (57.1)			
*Single father’s mother* *Single father’s father*	*14 (82.4)* *3 (17.6)*	*15 (97.75)* *1 (6.25)*			
Child’s babysitter	11 (35.5)	7 (25.0)			
Child’s uncle/aunt *Single father’s brother* *Single father’s sister*	3 (9.7) *1 (33.3)* *2 (66.7)*	5 (17.9) *0* *5 (100.0)*			
	*M (SD)*	*M (SD)*	*F* (df)	*p*	*η*_p_^2^
Child age (months)	95.68 (19.20)	100.00 (21.93)	0.652 (1,57)	0.423	0.011
Father age (years)	45.26 (6.71)	44.96 (7.01)	0.027 (1,57)	0.870	<0.001
Annual household income	70,032.26 (28,568.32)	65,357.14 (26,226.53)	0.426 (1,57)	0.517	0.007

Note: For 2 × 2 contingency tables, chi-square statistic with Yates correction was considered.

**Table 2:** Associations between children’s and fathers’ demographic factors, child attachment security, coparenting quality in fathers’ families of origin, observed coparenting, and observed family alliance, by family type (*N* = 59).

	1.	2.	3.	4.	5.	6.	7.	8.	9.	10.	11.
1. Child gender	1.00	−0.235	−0.091	−0.014	−0.415 *	−0.359 *	−0.232	−0.130	−0.102	−0.139	−0.051
2. Child age	0.040	1.00	0.213	0.433	0.258	0.381 *	0.449 *	−0.068	0.004	−0.011	−0.028
3. Number of siblings	0.265	−0.028	1.00	−0.080	−0.006	−0.129	0.054	−0.003	0.109	−0.203	−0.080
4. Father age	0.193	0.392 *	0.017	1.00	−0.041	0.319^†^	0.208	−0.204	−0.273	−0.099	0.023
5. Father educational attainment	−0.362 ^†^	0.158	0.000	0.026	1.00	0.164	0.140	−0.259	−0.181	0.139	−0.120
6. Annual household income	−0.135	0.196	−0.025	0.313	0.220	1.00	0.177	−0.069	−0.259	0.413 *	0.039
7. Child attachment security	0.067	0.396 *	0.202	0.284	0.161	0.186	1.00	0.368 *	0.279	−0.461 *	0.604 ***
8. Coparenting quality in the family of origin	−0.126	0.001	−0.053	0.132	0.031	0.098	0.158	1.00	0.212	−0.197	0.395 *
9. LTP supportive coparenting	−0.260	−0.453 *	0.161	−0.342 ^†^	0.189	−0.120	0.166	0.251	1.00	−0.333 ^†^	0.219
10. LTP conflictual coparenting	−0.167	−0.113	−0.081	−0.089	0.083	−0.109	−0.433 *	−0.444 *	−0.118	1.00	−0.339 *
11. Family alliance score	−0.050	0.376 *	−0.275	0.220	0.339 ^†^	0.383 *	0.544 **	0.242	−0.022	−0.211	1.00

Note: Associations for gay single-father families are displayed above the diagonal, whereas associations for heterosexual single-father families are displayed below the diagonal. Spearman *r* correlations were used for the associations between child gender and child age, number of siblings, father age, father educational attainment, annual household income, child attachment security, coparenting quality in the family of origin, LTP supportive coparenting, LTP conflictual coparenting, and family alliance; Pearson *r* correlations were used for the associations between child age, number of siblings, father age, father educational attainment, annual household income, child attachment security, coparenting quality in the family of origin, LTP supportive coparenting, LTP conflictual coparenting, and family alliance. Child gender is coded as: −1 = boy; 1 = girl. ^†^ *p* < 0.09. * *p* < 0.05. ** *p* < 0.01. *** *p* < 0.001.

**Table 3:** Family alliance assessment scale mean scores across family type and child gender (*N* = 59).

		Full Sample (*N* = 59)	Gay Single-Father Families (*n* = 31)	Heterosexual Single-Father Families (*n* = 28)				Boy (*n* = 31)	Girl (*n* = 28)			
		*M (SD)*	*M (SD)*	*M (SD)*	Wilks’ λ (16,40)	*p*	η_p_^2^	*M (SD)*	*M (SD)*	Wilks’ λ (16,40)	*p*	η_p_^2^
					0.727	0.536	0.273			0.739	0.590	0.261
					*F* (1, 55)	*p*	η_p_^2^			*F* (1, 55)	*p*	η_p_^2^
*Participation*	Postures and gazes	1.42 (0.67)	1.32 (0.75)	1.54 (0.58)	1.333	0.253	0.024	1.48 (0.63)	1.36 (0.73)	0.354	0.554	0.006
	Inclusion of partners	1.37 (0.67)	1.35 (0.71)	1.39 (0.63)	0.073	0.788	0.001	1.39 (0.67)	1.36 (0.68)	0.007	0.935	<0.001
*Organization*	Role implication	1.36 (0.64)	1.35 (0.66)	1.36 (0.62)	0.007	0.932	<0.001	1.45 (0.57)	1.25 (0.70)	1.425	0.238	0.025
	Structure	1.49 (0.57)	1.48 (0.57)	1.50 (0.58)	0.012	0.914	<0.001	1.48 (0.57)	1.50 (0.58)	0.012	0.914	<0.001
*Focalization*	Co-construction	1.32 (0.57)	1.35 (0.61)	1.29 (0.53)	0.178	0.674	0.003	1.32 (0.60)	1.32 (0.55)	0.000	0.989	<0.001
	Parental scaffolding	1.47 (0.63)	1.39 (0.67)	1.57 (0.57)	1.091	0.301	0.019	1.55 (0.57)	1.39 (0.69)	0.711	0.403	0.013
*Affect sharing*	Family warmth	1.46 (0.54)	1.48 (0.51)	1.43 (0.57)	0.206	0.652	0.004	1.45 (0.57)	1.46 (0.51)	0.000	0.988	<0.001
	Validation	1.46 (0.62)	1.45 (0.62)	1.46 (0.64)	0.016	0.901	<0.001	1.39 (0.67)	1.54 (0.58)	0.766	0.385	0.014
	Authenticity	1.51 (0.54)	1.61 (0.50)	1.39 (0.57)	2.402	0.127	0.042	1.52 (0.51)	1.50 (0.58)	0.049	0.826	0.001
*Timing/* *Synchronization*	Mistakes during activities	0.68 (0.68)	0.65 (0.71)	0.71 (0.66)	0.159	0.692	0.003	0.68 (0.65)	0.68 (0.72)	0.003	0.955	<0.001
	Mistakes during transitions	0.73 (0.72)	0.61 (0.67)	0.86 (0.76)	1.771	0.189	0.031	0.71 (0.69)	0.75 (0.75)	0.116	0.734	0.002
*Coparenting*	Support	1.25 (0.68)	1.26 (0.73)	1.25 (0.69)	0.030	0.864	0.001	1.35 (0.71)	1.14 (0.65)	1.448	0.234	0.026
	Conflict	0.64 (0.71)	0.65 (0.71)	0.64 (0.73)	0.015	0.904	<0.001	0.74 (0.73)	0.54 (0.69)	1.227	0.273	0.022
*Child contribution*	Involvement	1.20 (0.58)	1.16 (0.58)	1.25 (0.59)	0.174	0.679	0.003	1.35 (0.49)	1.04 (0.64)	4.340	0.042	0.073
	Goal-directed partnership	1.32 (0.57)	1.29 (0.53)	1.36 (0.62)	0.228	0.635	0.004	1.32 (0.60)	1.32 (0.55)	0.005	0.946	<0.001
Family alliance—total score		15.51 (4.13)	15.65 (4.35)	15.36 (3.95)	0.081	0.777	0.001	15.65 (4.25)	15.36 (4.07)	0.081	0.777	0.001

Note: The family alliance continuous score was calculated considering the 11 LTP subscales of participation, organization, focalization, affect sharing interactive functions, and timing/synchronization dimensions.

## Data Availability

The datasets generated for this study are available upon reasonable request to the corresponding author.
